# Genomic selection for recovery of original genetic background from hybrids of endangered and common breeds

**DOI:** 10.1111/eva.12113

**Published:** 2013-10-14

**Authors:** Carmen Amador, Ben J Hayes, Hans D Daetwyler

**Affiliations:** 1 INIAMadrid, Spain; 2 Department of Environment and Primary Industries, Biosciences Research DivisionBundoora, Vic., Australia; 3 La Trobe UniversityBundoora, Vic., Australia; 4 Cooperative Research Centre for Sheep Industry InnovationArmidale, NSW, Australia

**Keywords:** de-introgression, original background recovery, undesired introgression

## Abstract

Critically endangered breeds and populations are often crossed with more common breeds or subspecies. This results in genetic admixture that can be undesirable when it challenges the genetic integrity of wild and domestic populations, causing a loss in special characteristics or unique genetic material and ultimately extinction. Here, we present two genomic selection strategies, using genome-wide DNA markers, to recover the genomic content of the original endangered population from admixtures. Each strategy relies on the estimation of the proportion of nonintrogressed genome in individuals based on a different method: either genomic prediction or identification of breed-specific haplotypes. Then, breeding programs that remove introgressed genomic information can be designed. To test these strategies, we used empirical 50K SNP array data from two pure sheep breeds, Merino (used as target breed), Poll Dorset and an existing admixed population of both breeds. Sheep populations with varying degrees of introgression and admixture were simulated starting from these real genotypes. Both strategies were capable of identifying segment origin, and both removed up to the 100% of the Poll Dorset segments. While the selection process led to substantial inbreeding, we controlled it by imposing a minimum number of individuals contributing to the next generation.

## Introduction

Cross-breeding is a strategy commonly used in livestock and occurs naturally in many wild populations. The increase in fitness that a new genetic input can infuse has been widely studied, and mixing breeds have been used in livestock to increase genetic variability and the performance for productive traits (Frankham et al. 2002; Schaeffer et al. 2011). Nevertheless, mixing genetic pools can also have disadvantages (Rhymer and Simberloff 1996; Allendorf et al. 2001). For example, admixture between wild and cultivated salmon is thought to result in fitness reduction in the indigenous populations and loss of local adaptations (Araki et al. 2007; Ford and Myers 2008). Some wild populations are endangered because domestic relatives live in close proximity, causing a loss of biodiversity through introgressive hybridization (Randi 2008). Examples of this have been described in a variety of species such as cattle (Padilla et al. 2009), partridge (Barbanera et al. 2011), trout (Hohenlohe et al. 2011), mink (Cabria et al. 2011) and salamander (Bayer et al. 2012).

In livestock, cross-breeding local populations with more productive breeds has in some cases led the first to lose their specific characteristics and adaptive traits such as disease resistance, adaptation to a specific climate or harsh conditions (Taberlet et al. 2008). This threatens some of these breeds, and their original genetic background should be recovered in order to avoid extinction (Ugarte et al. 2001; Morais et al. 2005; Taberlet et al. 2008). For example, Fribourg Black and White cattle, a Swiss dual-purpose breed quite phenotypically similar to Simmental cattle, have become extinct due to continuous crossing with Holstein cattle. It is clear that in some situations, an effective approach to remove exogenous genetic material would be desirable to recover the original genetic background of such populations.

The process of recovering the genome of a population that has suffered undesired introgression is called de-introgression. Previous strategies for de-introgression have proceeded by minimizing the genealogical coancestry of candidates with the exogenous individuals entering the population (Amador et al. 2011), minimizing the molecular coancestry calculated through genome-wide information (50 000 SNP) (Amador et al. 2013), or identifying probability of origin with multiallelic markers through measures of differentiation (Amador et al. 2012).

Removing exogenous material using only genealogical coancestry obtained good results in several simulated scenarios. However, it required a completely recorded pedigree, which is unlikely to exist in wild populations (Amador et al. 2011). Minimizing the molecular coancestry calculated through genome-wide information provided the best results regarding the amount of native genome recovered (Amador et al. 2013). The success in the recovery when using other kinds of markers depended on the number of markers and allele frequencies. Hence, exclusive markers (i.e. having private alleles occurring only in native or exogenous populations) obtained good results even with few markers (5–10), and more markers were required when alleles were segregating in both native and exogenous populations at intermediate frequencies (i.e. more similar populations). The number of markers available was a major limiting factor and essential to choose the appropriate method when facing the task of recovering the desired background (Amador et al. 2012). Moreover, this approach requires a perfect knowledge of the allele frequencies of the original pure populations. A side effect of all three methods was rapid increase in inbreeding due to the restriction in the number of individuals contributing to the next generations.

Studies on genomic selection have proven that genome-wide information can be used for identifying those fragments of the genome more related with productive characters. Genomic selection methods have also been used for prediction of breed composition in cattle (Frkonja et al. 2012). Our aim here was to modify two genomic approaches to identify and recover chromosome segments of endangered breeds, when undesired introgression has mixed their background with other breeds.

The first method was originally developed in cattle to differentiate between segments of *Bos indicus* and *Bos taurus* origin by Bolormaa et al. (2011). The second method, adapted from VanRaden et al. (2011), used a linear model to predict breed identity through the genomic relationship matrix between pure and crossed individuals. Several scenarios of admixture were simulated using real genotypes as a base population. The simulated genotypes were used to test the ability of the two mentioned methods to remove the foreign genetic information from a mixed population. A real data set of 6000 sheep was used. The genotypes (OvineSNP50 BeadChip) of individuals of two pure breeds (Merino and Poll Dorset) and F1 crosses of these breeds were used to evaluate the ability of two approaches to determine the level of similarity of the crosses to the pure breeds.

The first objective of this work was to analyse the efficiency of these two methods to detect Merino versus Poll Dorset segments in real F1 crossbred individuals. The second objective was to evaluate the efficiency of the methods in breeding programs to restore full Merino background after introgression events of varying magnitude by removing individuals carrying Poll Dorset genetics, as well as comparing these results with those obtained in previous studies. The Merino breed is not endangered, and the crosses with Poll Dorset sheep are intentional (and for productivity purposes). However, the data set is useful for evaluating the performance of our strategies.

## Material and methods

### Data set

The data used was obtained from the Australian Cooperative Research Centre for Sheep Industry Innovation (Sheep CRC) (Rowe 2010; van der Werf et al. 2010). Individuals from three populations were used: 4964 pure Merino, 188 pure Poll Dorset and 811 crosses (all of them 50% Merino and 50% Poll Dorset). The pedigree of the individuals was available and used to calculate the genealogical coancestries.

The individuals were genotyped using the 54 977 single-nucleotide polymorphisms (SNP) included in the OvineSNP50 BeadChip (Illumina, San Diego, CA, USA). The quality control measures applied were the following: SNP were removed if they had a call rate of less than 95%, a minor allele frequency of less than 0.01, a GenCall score of less than 0.6. The SNP were also removed if they were out of Hardy–Weinberg equilibrium (a *P*-value cut-off of 10–15), had no genome location or their LD with another SNP on the chip were greater than 0.99. After the quality control, 48 599 SNP were used. Data for genotyped animals were removed if their genotype call rate was <0.9 or their mean heterozygosity was greater than 0.5, which would indicate sample contamination. Sporadic-missing SNP were imputed using Beagle (Browning and Browning 2009). The genotypes were phased using ChromoPhase (Daetwyler et al. 2011).

### Breed origin prediction

Two methods were implemented to predict breed proportion of Merino and Poll Dorset.

#### Haplotype approach

This method, described by Bolormaa et al. (2011), relies on haplotype frequencies in both original breeds and requires phased genomic data. Each chromosome was divided into nonoverlapping segments consisting of 10 consecutive SNP defining the length of haplotypes. We estimated the probability of a segment *i* being of Merino origin (*b*_*Mi*_) for each of the up to 2^10^ possible haplotypes for a specific segment as:


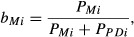
(1)

where *p*_*Mi*_ is the frequency of the *i*th haplotype in the pure Merino animals and *p*_*PDi*_ is the frequency of the *i*th haplotype in the pure Poll Dorset animals. All haplotypes were classified as either Merino or Poll Dorset in origin based on their *b* values. Haplotypes with a *b* value lower than 0.4 were classified as Poll Dorset and those with a *b* value higher than 0.6 were classified as Merino. The remaining haplotypes were left unassigned because their breed origin was ambiguous. The overall proportion of an animal's genome that is of Merino origin was calculated as the number of Merino assigned haplotypes divided by the total number of haplotypes.

#### Genomic selection approach (GBLUP)

Genomic selection models have been successfully used to predict additive genetic values for production, health and welfare traits in many domestic species (Hayes and Goddard 2008; Hayes et al. 2009a; Daetwyler et al. 2010; Wolc et al. 2011), and several methods have been proposed (de los Campos et al. 2013). All genomic selection methods make use of a reference population with phenotypes and genotypes to predict individuals with only genotypes, usually young selection candidates (Daetwyler et al. 2013). In this study, we apply the widely used genomic selection method genomic best linear unbiased prediction (GBLUP) to predict the *Merino proportion* of the admixed individuals. In other words, the phenotype was the *Merino proportion* of each animal, as determined by pedigree records. The pure Merino and pure Poll Dorset animals were used as the reference population with phenotypes coded 1 and 0, respectively. Then, the *Merino proportion* was predicted in individuals with only genotypes of the three populations (pure Merino, pure Poll Dorset and crossbred individuals). The GBLUP approach was adapted from VanRaden et al. (2011), and the following model was fitted in the statistical software ASReml (Gilmour et al. 2009):


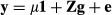
(2)

where **y** is a vector with the proportion of Merino of the animals (as the dependent variable), *μ* is the intercept, **1** is a vector of ones, **Z** is a incidence matrix relating additive genetic effects to phenotypes, **g** is a vector of additive genetic effects, and **e** is the vector of residuals. The following distributions were assumed: **g** ~ *N* (0, **G***σ*^2^_*g*_) and **e** ~ *N* (0, **I***σ*^2^_*e*_). **G** was a genomic relationship matrix, calculated as done in Yang et al. (2010), which measures allele sharing between individuals. Our use of **G** allows direct prediction of breed proportion, whereas VanRaden et al. (2011) predicted marker effects from which breed proportions were calculated. Both models have been shown to be equivalent (Habier et al. 2007; Goddard 2009). A heritability of 99% was assumed for the trait *Merino proportion*.

### Testing strategies in real data

The haplotype and GBLUP approaches were tested in the real data sets with both genotypes and known breed composition to predict the proportion Merino in Merino, Poll Dorset and F1 crossbred individuals.

### De-introgression simulation

#### Creating introgressed populations

**Figure 1:**
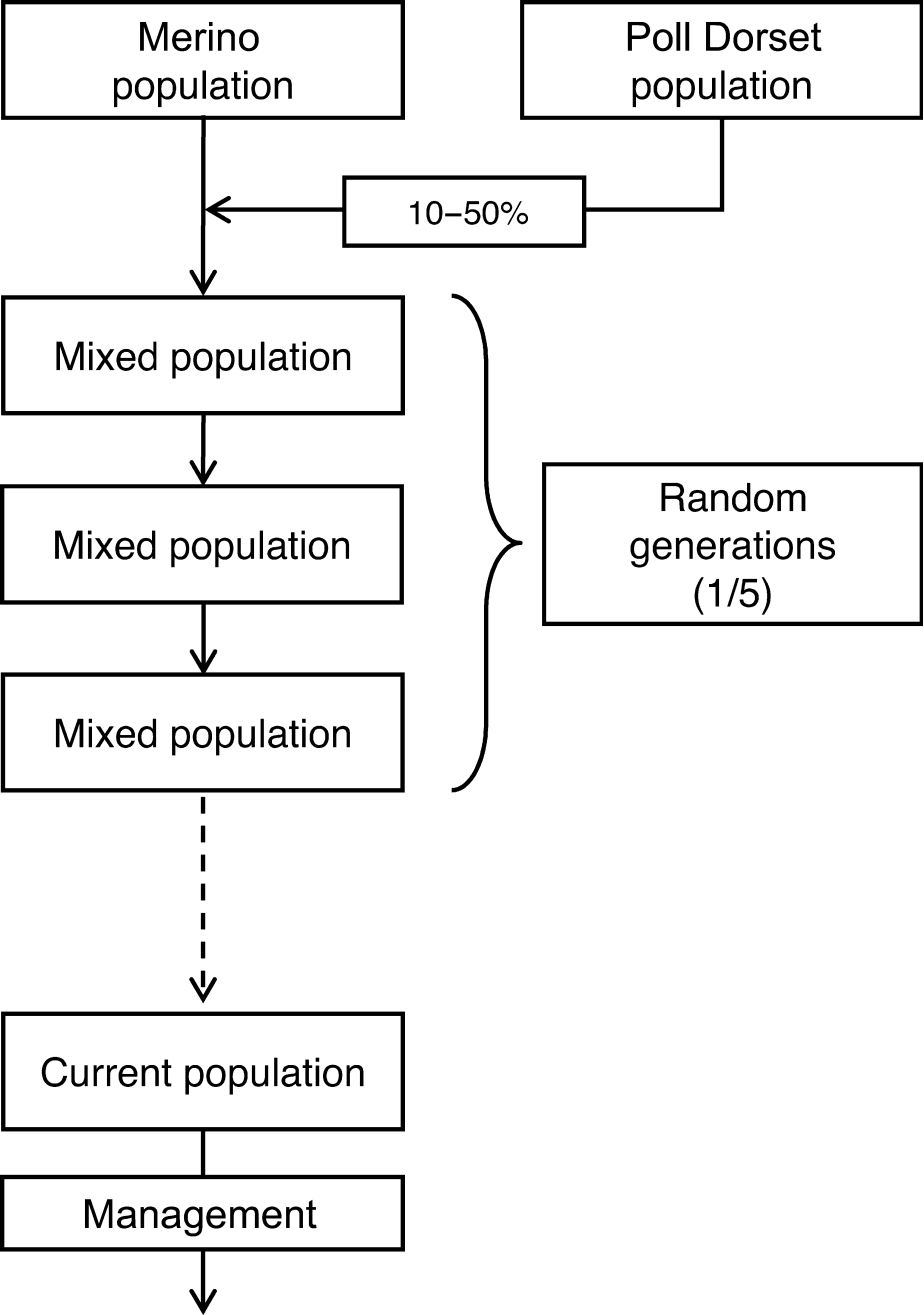
Diagram of the introgression simulation. The real individuals were used to create a mixed population of 100 individuals that mated randomly during one to five generations. Next, five generations of management started.

The pure Merino and pure Poll Dorset genotypes (see data set section) were used to simulate several introgression scenarios from which the Merino genetic background was intended to be recovered (Fig. 1). A population of 100 individuals was created using the real individuals, with a variable number of Poll Dorset (10, 20, 30, 40 or 50) and the rest (up to 100) being Merino.

The sheep genome is made up of 26 autosomes. Each chromosome was considered to be 1m, and to create gametes, a Poisson distributed (λ = 1) number of crossingovers (one crossover is expected on average per Morgan) with no interference were generated in random positions over the chromosome.

Sex was randomly assigned to the genotypes used to create the individuals (50 males and 50 females). The mixed population mated randomly during one, three or five generations to produce different levels of admixture of the Poll Dorset genetic information into the Merino background. Five generations of management were then simulated using two different approaches to remove the Poll Dorset genetics. The real origin of the alleles was known in all generations and was used to evaluate the efficiency of recovery. The pedigree was also known and used for calculating coancestries and inbreeding. Twenty replicates per scenario were simulated.

#### Recovery of original genetic background

Once the mixed populations comprising 100 individuals were created, we selected the purest Merino individuals in order to recover the largest number of Merino alleles over several generations. For that purpose, a group of pure individuals from the original Merino and Poll Dorset populations was used as reference for the following approaches:

##### Haplotype approach

A random sample of 188 pure Merinos and all 188 pure Poll Dorset genotypes were used for training the haplotype approach at the beginning of each replicate. In the following generations, haplotype *b* values were computed for all available individuals using eqn (1), and a mean *b* value per individual was calculated. Those individuals with largest mean *b* values were assumed to be the purest Merinos.

##### GBLUP approach

A random sample of 188 pure Merinos (the same subset as the haplotype approach each replicate) and the 188 pure Poll Dorset genotypes were included with known breed (i.e. phenotypes), together with the genotypes for the selection of candidates in each generation. Breed proportions were then predicted using model (2). The predictions were used to identify the purest Merino animals in each generation of management.

Exogenous information was eliminated in each generation of management by choosing the 10 purest Merino animals per sex (i.e. the 20 individuals with highest number of Merino haplotypes) to equally contribute to the next generation (10 offspring each). Individuals were mated randomly. The above procedure implies a theoretical rate of inbreeding per generation (*ΔF*) of 0.0125 (assuming random mating).

#### Evaluation of strategies

In every generation, two variables were calculated to evaluate the efficiency of the strategies: (i) percentage of Merino (*Merino proportion*) (i.e. the real proportion of alleles coming from Merino founders) and (ii) inbreeding coefficient (*F*). The *F* values were calculated considering the real coancestries between the original individuals (from the real pedigree) and the genealogy of the admixture and management periods. From these, values of *ΔF* were calculated as:


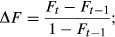
(3)

where *t* is the number of the current generation (one to five) (Falconer and Mackay 1996).

#### More extreme introgression scenarios

Extra simulations were carried out to cover more extreme situations, including a longer period of admixture before de-introgression and lower selection pressure during de-introgression (i.e. forcing more individuals to contribute thereby lowering rate of inbreeding). The number of individuals actually contributing was increased to 40 (i.e. 20 males and 20 females, five offspring each) in scenarios with five and 20 generations of admixture, to assess the rate of removal of Poll Dorset genetic material that was accomplished.

#### Different SNP used for de-introgression and evaluation

The previous scenarios constitute the upper bound of efficiency as de-introgression is tested in the same loci used for the management. We also investigated the recovery of ungenotyped loci by only using half of the markers for de-introgression and evaluated the efficiency using the other half in the scenario with 20 generations of admixture (40 individuals contributing). The number of markers in a haplotype was 10, as in the other scenarios, and alternate SNP were masked.

## Results

### Evaluation of breed proportion estimation

#### Haplotype approach

**Figure 2:**
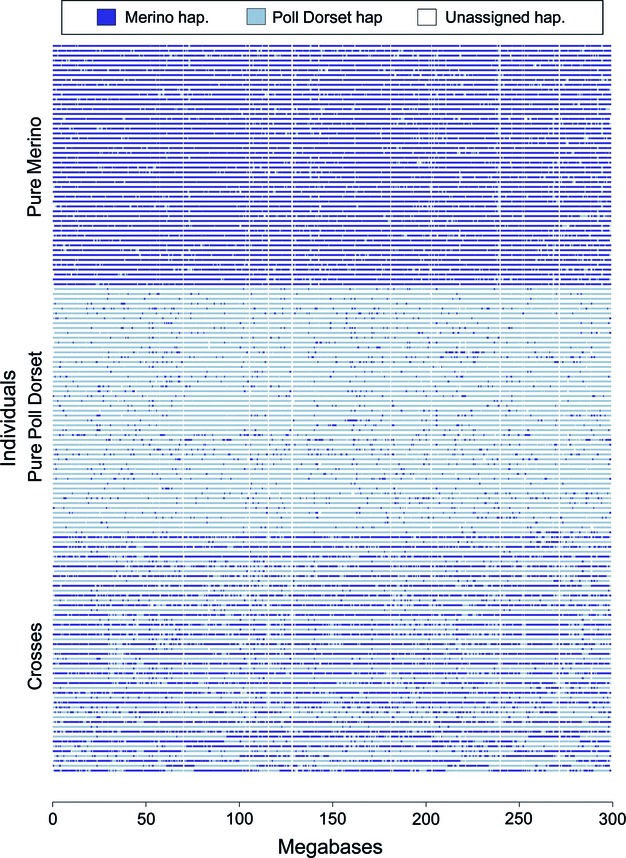
Plot of the 549 haplotypes origin of chromosome 1 in a sample of 25 random pure Merino, 25 random pure Poll Dorset and 25 random crossed animals. Each line represents an individual's chromosome.

The distribution of the haplotypes in chromosome 1 of 25 randomly sampled pure Merinos, 25 randomly sampled pure Poll Dorset and 25 randomly sampled crossed individuals is shown in Fig. 2. In the figure, each line shows one chromosome, and thus, each individual is represented by two consecutive lines. The proportion of unassigned haplotypes was 0.049 ± 0.001 in all the groups, and most of the segments were correctly classified (91%). The distribution of the type of segments in the crossed individuals agrees with all of them being F1 crosses because they have one entire chromosome coming from Merino and the other one coming from Poll Dorset.

As stated before, most of the segments in the Merino group were recognized as Merino (90% of the segments with a *b* value >0.6) and most of the segments in the Poll Dorset group are recognized as Poll Dorset (91% of the segments with a *b* value <0.4). The results in the crossed animals showed a mixed pattern with 58% of the segments considered Poll Dorset and 37% of the segments considered Merino. The higher number of Poll Dorset segments in the crosses could suggest that the Poll Dorset individuals are not as pure as expected. Thus, some Merino segments, present in Poll Dorset individuals are recognized as Poll Dorset. The distribution of the *b* values of the 10 SNP segments in the three groups of individuals (across the entire genome) is shown in Fig. S2.

#### GBLUP

**Figure 3:**
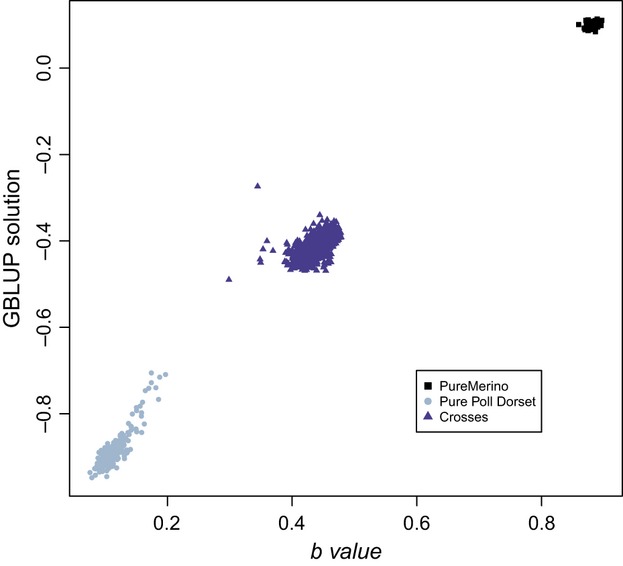
Comparison of the GBLUP solutions and the haplotypes results. The GBLUP solutions are represented on the vertical axis and the mean *b* values per individual from the haplotype approach on the horizontal axis.

The classification of individuals from the GBLUP genomic prediction of *Merino proportion* is shown on Fig. 3 compared with the mean *b* value of each individual. Data show that the GBLUP method is also able to separate the three groups of individuals. The correlation between the GBLUP solution and the mean *b* values was 0.99 (in the full data set).

### Evaluation of de-introgression efficacy

Results for the percentage of Merino recovered for the haplotype approach and GBLUP approach were very similar in all scenarios; consequently, only the results for the haplotype approach are presented.

**Figure 4:**
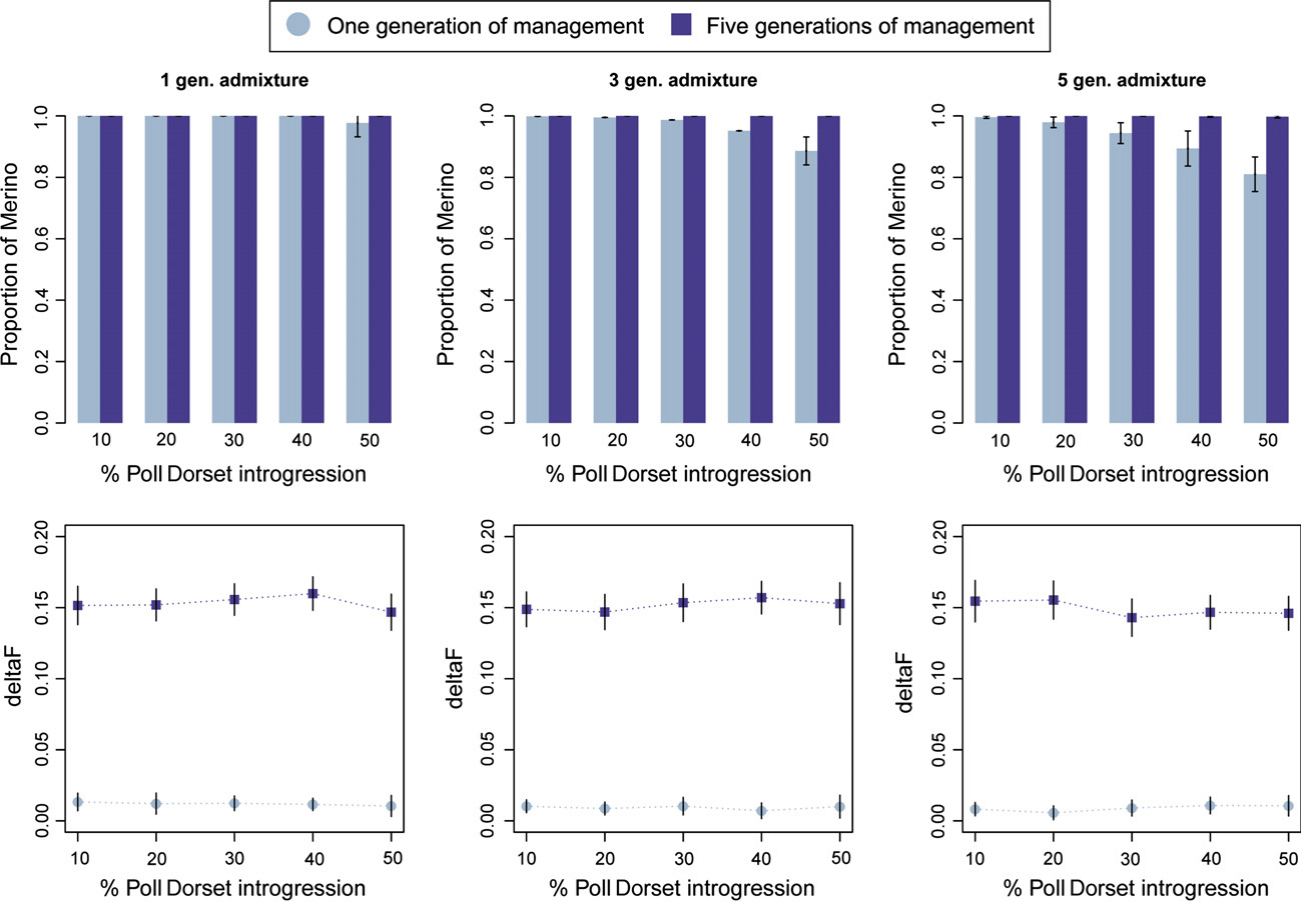
Proportion of Merino recovered (upper panels) after one or five generations of management and *ΔF* (lower panels) after one or five (cumulative *ΔF)* generations of management using the haplotype approach, for one, three or five generations of admixture (20 individuals contributing).

The values of *Merino proportion* obtained after one or five generations of management in the different introgression scenarios are shown in Fig. 4 (upper panel). The Merino background was almost completely recovered in all the scenarios after five generations of breeding. The recovery was nearly completed in the first generation of breeding when the percentage of Poll Dorset introgression was small and only mixed for one or three generations, but it took longer in the scenarios with more generations of admixture prior to the de-introgression.

The results of inbreeding reached after managing one or five generations with the haplotype approach are shown in Fig. 4 (lower panel). The values of *ΔF* obtained were close to the expectation (*ΔF*_*1*_ = 0.01) in the first generation of management, but they became higher than the expectations in the subsequent generations (*ΔF*_*2, 3, 4, 5*_ = 0.035). This is because *ΔF* was calculated assuming random selection and mating in the parents. Selecting the purest individuals resulted in individuals that were more related than if they were selected at random after the second generation of management.

The results of inbreeding were slightly different when using the GBLUP approach (Fig. S3). The accumulated *ΔF* after five generations of management is lower when using GBLUP (*ΔF*_hap_* = *0.15, *ΔF*_GBLUP_* = *0.13). The differences in *ΔF* appeared in the final generations, once the maximum *Merino proportion* was achieved, but the de-introgression method was still being applied. If estimated proportions are similar for all available individuals, the selected breeding animals are chosen at random not involving a higher level of relationship between them, and thus, *ΔF* is closer to expectations. In contrast, the haplotype-based method still detected differences between candidates related to common origin leading to higher *ΔF*.

**Figure 5:**
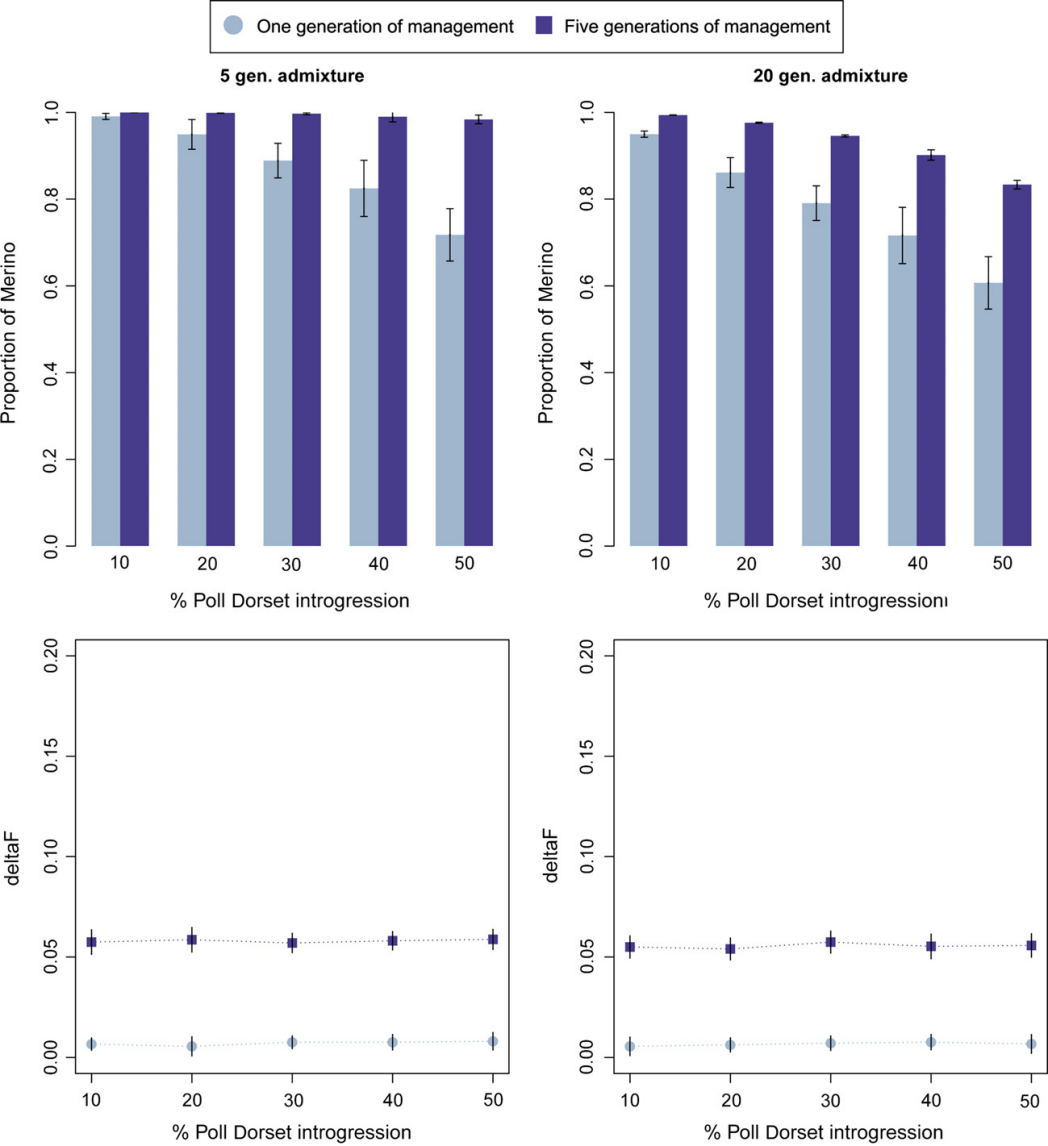
Proportion of Merino recovered (upper panels) after one or five generations of management and *ΔF* (lower panels) after one or five (cumulative *ΔF)* generations of management using the haplotype approach for five or 20 generations of admixture (40 individuals contributing).

Results obtained when doubling the number of contributing individuals to 40 (haplotype approach) for five generations of admixture are shown in Fig. 5 (left). The proportion of Merino recovered in these simulations was considerably lower for one generation of breeding, due to the smaller selective pressure. Consequently, the method requires the five generations of breeding to achieve the maximum recovery but with a smaller increase in inbreeding.

A more extreme situation was tested in which exogenous material was admixed for a longer period of 20 generations. In this scenario, the number of breeding individuals during de−introgression was also 40. The results (Fig. 5, right) demonstrate that the method can use the haplotype information even when the admixture time is long and reach up to an 80% of Merino in five generations of management with an acceptable level of inbreeding. As observed above, the *ΔF* in the first generation of breeding is close to the expectation, but it is higher in the following generations of breeding.

The results obtained when using different SNP for de-introgression and evaluation are shown in Table 1 for one generation of management in a scenario with 20 generations of admixture (10–50% of introgression, 40 individuals contributing), together with those obtained using all the SNP for both tasks. De-introgression efficiency with this reduced SNP set was very similar to that obtained when using all SNP.

**Table 1:** Proportion of *Merino recovery* and *ΔF* obtained after one generation of de-introgression in scenarios with 20 generations of admixture (40 individuals contributing) using different SNP markers for de-introgression and evaluation.

	All SNP used for de-introgression and evaluation				
% Poll Dorset	10	20	30	40	50
*Merino Recovery*	0.950 ± 0.031	0.861 ± 0.059	0.794 ± 0.074	0.716 ± 0.105	0.607 ± 0.095
*ΔF*	0.006 ± 0.005	0.006 ± 0.004	0.007 ± 0.004	0.008 ± 0.004	0.007 ± 0.005

	Different SNP used for de-introgression and evaluation				
% Poll Dorset	10	20	30	40	50

*Merino Recovery*	0.946 ± 0.030	0.860 ± 0.058	0.794 ± 0.073	0.714 ± 0.103	0.605 ± 0.097
*ΔF*	0.007 ± 0.006	0.006 ± 0.005	0.006 ± 0.005	0.007 ± 0.005	0.007 ± 0.005

## Discussion

Many populations are close to extinction due to genetic introgression. Our results demonstrate that some of this introgression could be reversed by detecting and breeding those individuals containing chromosome segments of the original population.

In the present study, we used a real sheep data set to demonstrate the ability of two methods to find out the origin of genetic information in an admixed population from two different breeds, even after several generations of introgression. Results showed that both methods were able to detect the proportion of Merino and Poll Dorset with high accuracy. The correlation between the prediction of the Merino content using GBLUP and *b* values (from the haplotype method) was 0.99 in the whole population, but just 0.54 when calculated for the crossed individuals. Despite this medium correlation, there was a clear tendency of higher *b* values implying higher GBLUP solutions (Fig. S4). This was confirmed when evaluating their performance in the de-introgression process. The degree of Merino recovered was the same for both methods, suggesting that they identify almost the same individuals as the purest Merinos.

With respect of the number of generations effectively removing the Poll Dorset haplotypes, the performance was very similar to what was observed in previous studies (Amador et al. 2011, 2012). Most of the recovery was achieved in the first generation of management, but often the most admixed scenarios required one or two more generations. Besides, the strategy implied an increase in inbreeding due to the reduced number of individual contributing.

The GBLUP and haplotype approaches both recovered more genome of the target breed than pedigree (Amador et al. 2011) or microsatellite-like markers methods (Amador et al. 2012). It is expected that using genome-wide information outperforms the use of the pedigree because the latter gives average expected values while the former provides the particular realizations in every region of the genome in linkage disequilibrium with the markers (Hayes et al. 2009b; de Cara et al. 2011). The results obtained in the present study were also much better in the removal of exogenous alleles than those obtained by minimizing the molecular coancestry calculated from 50 000 SNP on simulated genomes (i.e. also using genome-wide information) (Amador et al. 2013). This suggests that the GBLUP and haplotype approaches could be better than minimization of molecular coancestry. However, the analysed data sets differed between the two studies. While the molecular coancestry approach was tested on simulated data, the GBLUP and haplotype approach here have been tested on real data. The number of SNP in both studies was similar, and the simulated and real data set only changed in the similarity between the native and exogenous population. Also, when measuring de-introgression efficiency using a reduced number of SNP and evaluating the results in a different set, the results were equally successful, showing that the removal of exogenous alleles is also effective in ungenotyped regions of the genome.

A key to the achievement of these kinds of de-introgression is the differentiation between the target population for recover and the population it has admixed with. If the admixed population are genetically similar to the target population, then it is very difficult to differentiate between both genetic backgrounds, and thus, de-introgression is more difficult to achieve. If both populations are clearly different, the task is easier. In our data set, with hard conditions, recovery was successful in a few generations of management. Even after 20 generations of admixture, the methods are still capable of recognizing the Merino or Poll Dorset segments, proving that the populations are genetically different. The differences in performance between this study and Amador et al. (2013), where the recovery was also based on genome-wide data, may be partly due to the latter study's fully simulated data having more similar populations leading to more difficulty to recover the original background.

The current methods heavily rely on a group of genotyped pure individuals to train *b* values and GBLUP predictions. The haplotype approach cannot be applied without some native and exogenous pure individuals to evaluate the frequencies of segments in both original populations. If some pure samples from dead animals would be available, the method could be trained on ancient DNA. The GBLUP approach requires also two groups of individuals with different and known native proportions, but not necessarily the pure subsets. These could be a few pure individuals and some mixed individuals (provided the proportion of native genome is known, e.g. F1s). We tested the performance of Merino prediction through GBLUP using 188 pure Poll Dorset and 20 crossed individuals from the original data set as the reference population. The efficiency of the method did not decrease, obtaining similar results as in the whole reference data set (Table S1).

We also compared the results with those obtained in equivalent scenarios using the program Structure (Pritchard et al. 2000) to evaluate the *Merino proportion* of individuals and mate the purest for de-introgression. We obtained very similar results when using Structure (Table S2), but the software took much longer to evaluate the *Merino proportion* in the individuals (GBLUP and haplotypes approach: up to 5 min per chromosome and replica; Structure: up to 24 h per chromosome and replica).

We chose to include 10 markers in our haplotypes approach. Other methods (e.g. Lawson et al. 2012) can potentially define chromosomal ancestry break points more precisely. This could be helpful for de-introgression in highly admixed populations. Furthermore, mating schemes could be designed to reconstruct the original population genome via selection of individuals with complementary haplotypes.

In some other situations, the de-introgression process could be focused on some specific traits (e.g. related to local adaptation or phenotypic characteristics specific to a breed). In that case, molecular markers could help identify those traits and focus the restoration effort in keep them and maintain the rest of the genome. Also, phenotypes can be used directly for this purpose as in the study by Fernández et al. (2012), to achieve the recovery of regions of the genome linked to morphological traits. However, this is a much more difficult task than that attempted here, as a large number of phenotyped and genotyped individuals would likely be required.

The time elapsed between the admixture events and the start of breeding for recovery is also an important factor to take into account for the possibilities of recovery. The degree of Merino recovered after a longer period of admixture was noticeably smaller as shown in Fig. 5. This was also observed in other de-introgression studies (Amador et al. 2011, 2012).

When the admixture is not recent, more haplotypes will remain unassigned when using the same number of SNP and haplotype length, because the length of the introgressed haplotypes will decrease due to recombination in each generation. This will lead to individuals that cannot be identified as Merino or Poll Dorset for both strategies, and successful de-introgression will require a denser marker map. This highlights the importance of acting quickly to control introgression events. We observed that after 20 generations of admixture, some recovery can be achieved, but if the unmanaged period is too long, this recovery will be less effective.

The methods here described are designed for populations on which a SNP chip has been developed. The availability of SNP chip data in domestic species is increasing, especially due to genomic selection studies (Meuwissen et al. 2001), and panels of up to 770K are available in cattle (Lenstra et al. 2012). This density of markers is not available in wild species; nevertheless, new approaches for discovering SNP are being developed thanks to next-generation sequencing (Davey et al. 2011). Methods such as reduced-representation libraries (RRLs) and genotyping-by-sequencing (GBS) can be used in species without a reference genome, for SNP discovering on a small-scale, being cheaper and more feasible methods than developing SNP arrays (Van Tassell et al. 2008; Elshire et al. 2011).

On the other hand, SNP arrays are usually biased towards higher frequency SNP (i.e. ascertainment bias) and are developed using only a subset of the total diversity of different breeds. This bias could affect the results of a de-introgression process, especially if one of the breeds was not involved in the development of the SNP array, because markers will be more informative for one breed than for the other. It could also affect de-introgression efficiency of ungenotyped low-frequency alleles, because their LD with the higher frequency SNP on the array would be limited. In our case, the purest Merino individuals were still recognized despite potential SNP chip biases, evidenced by the efficient de-introgression. However, it is important to consider how the choice of markers may affect the estimation of breed proportion.

The increase in inbreeding every generation of management is a side effect of the methods due to the reduction in the number of individuals contributing to the next generation. Both GBLUP and the haplotypes approach implied a similar increase in inbreeding due to the de-introgression process. It cannot be avoided, but it can be controlled. As shown, increasing the number of individuals contributing decreases *ΔF* and still allows for the removing of exogenous information. Increasing the number of individuals selected to contribute, or setting an explicit restriction on *ΔF* could lead to an acceptable recovery without losing too much genetic diversity, but it has to be assessed in each particular situation and decide if this increase in inbreeding is acceptable to obtain a certain recovery of the native genetic alleles.

We have demonstrated that de-introgression of exogenous genome segments is feasible. The parameters simulated cover a wide range of situations and altering parameters will influence outcomes. For example, selecting 10 contributing individuals may not be feasible in a very small population. The expected effect on inbreeding levels must be evaluated with regard to speed (i.e. number of generations) and numbers of contributors to decide whether it is desirable to de-introgress.

## Data archiving statement

The real Ovine 50K SNP genotypes of the original 188 Merino and 188 Polled Dorset individuals and their breed classification are shared in the *Dryad Digital Repository*: http://dx.doi.org/10.5061/dryad.mn0d5.

## Supporting Information

**Figure S1**. Results of the principal component analysis. **Figure S2**. Distribution of the *b* values for 10 SNP segments across the 26 ovine chromosomes in pure Merino, pure Poll Dorset and F1 crossed individuals. **Figure S3**. Proportion of *Merino recovery* (upper panels) after one or five generations of management and ΔF (lower panels) after one or five (cumulative ΔF) generations of management using the GBLUP approach, for one, three or five generations of admixture (20 individuals contributing). **Figure S4**. Comparison of the GBLUP solutions and the haplotypes results in the crossed individuals. **Table S1**. Proportion of *Merino recovery* and ΔF obtained after one generation of de-introgression in scenarios with 20 generations of admixture (40 individuals contributing) using the GBLUP approach with a reference population of 188 Poll Dorset and 20 F1 crossed individuals from the original population. **Table S2**. Proportion of *Merino recovery* and ΔF obtained after one generation of de-introgression in scenarios with 20 generations of admixture (40 individuals contributing) using Structure (linkage model for *K* = 2 and default options).
