# Supplementary material for: Genomic selection for recovery of original genetic background from hybrids of endangered and common breeds
Source: Evol Appl. 2013 Oct 14;7(2):227–37. doi: 10.1111/eva.12113 (PMC3927885; doi:10.1111/eva.12113)
Supplement: Supplementary file 1 — Figure S1. Results of the principal component analysis. Figure S2. Distribution of the b values for 10 SNP segments across the 26 ovine chromosomes in pure Merino, pure Poll Dorset and F1 crossed individuals. Figure S3. Proportion of Merino recovery (upper panels) after one or five generations of management and ΔF (lower panels) after one or five (cumulative ΔF) generations of management using the GBLUP approach, for one, three or five generations of admixture (20 individuals contributing). Figure S4. Comparison of the GBLUP solutions and the haplotypes results in the crossed individuals. Table S1. Proportion of Merino recovery and ΔF obtained after one generation of de-introgression in scenarios with 20 generations of admixture (40 individuals contributing) using the GBLUP approach with a reference population of 188 Poll Dorset and 20 F1 crossed individuals from the original population. Table S2. Proportion of Merino recovery and ΔF obtained after one generation of de-introgression in scenarios with 20 generations of admixture (40 individuals contributing) using Structure (linkage model for K = 2 and default options). [file eva0007-0227-sd1.pdf]

Supporting information

A principal component analysis of the genomic relationship matrix (G) was performed using the R version 2.11.1 (R Development Core Team 2010) to prove the ability of the genomic information to differentiate between the three groups of individuals. Fig. S1 shows a plot of the first two principal components (those with larger eigenvalues). It can be observed that the first PC already separates the two breeds and the crosses. The crosses are situated half way between Merino and Poll Dorset groups. A further level of division can be observed within the Merino breed using the second PC. This differentiation is due to two large half-sib groups of Merino selection lines.

Fig. S1. Results of the principal component analysis. PC1 lies in the horizontal axis and PC2 in the vertical axis.

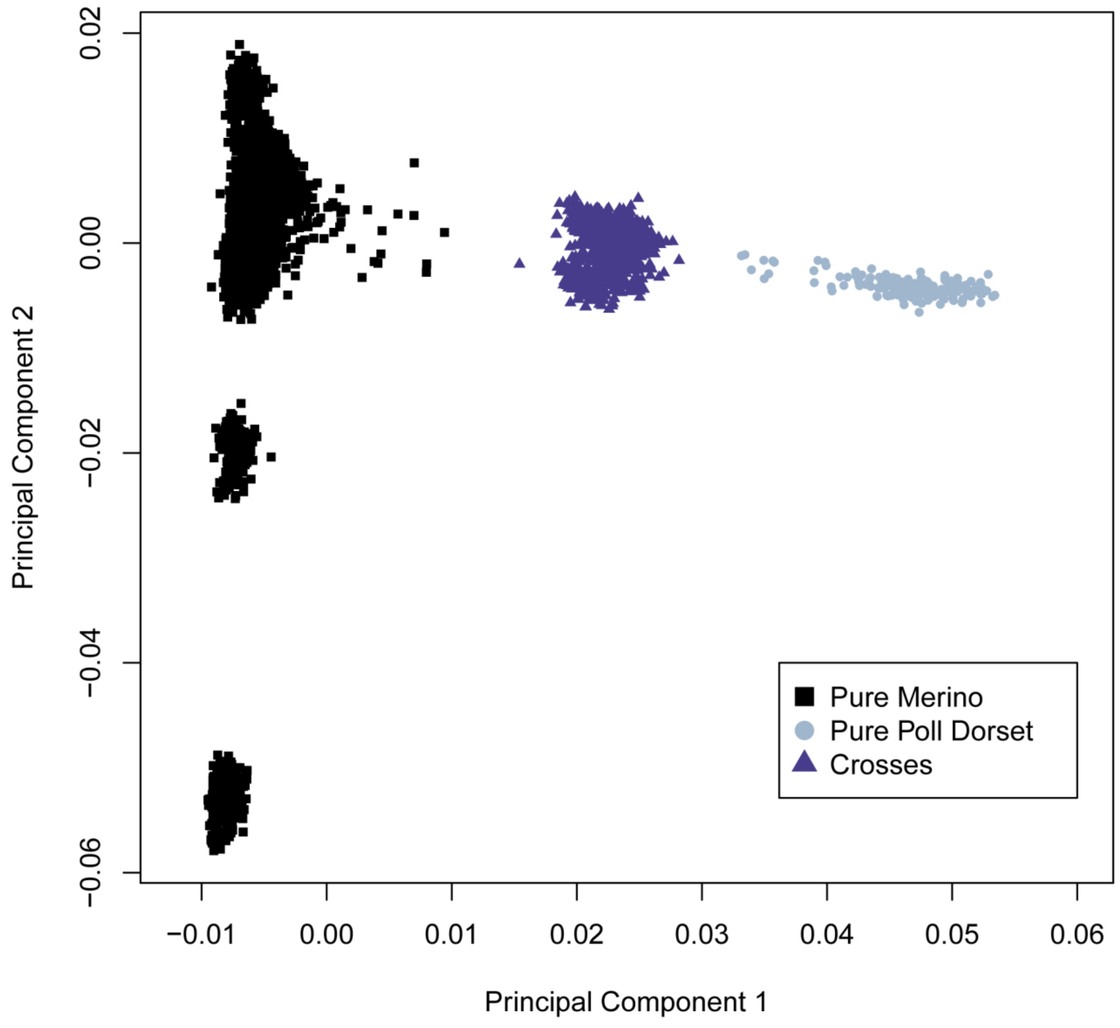

Fig. S2. Distribution of the b values for 10 SNP segments across the 26 ovine chromosomes in pure Merino, pure Poll Dorset and F1 crossed individuals.

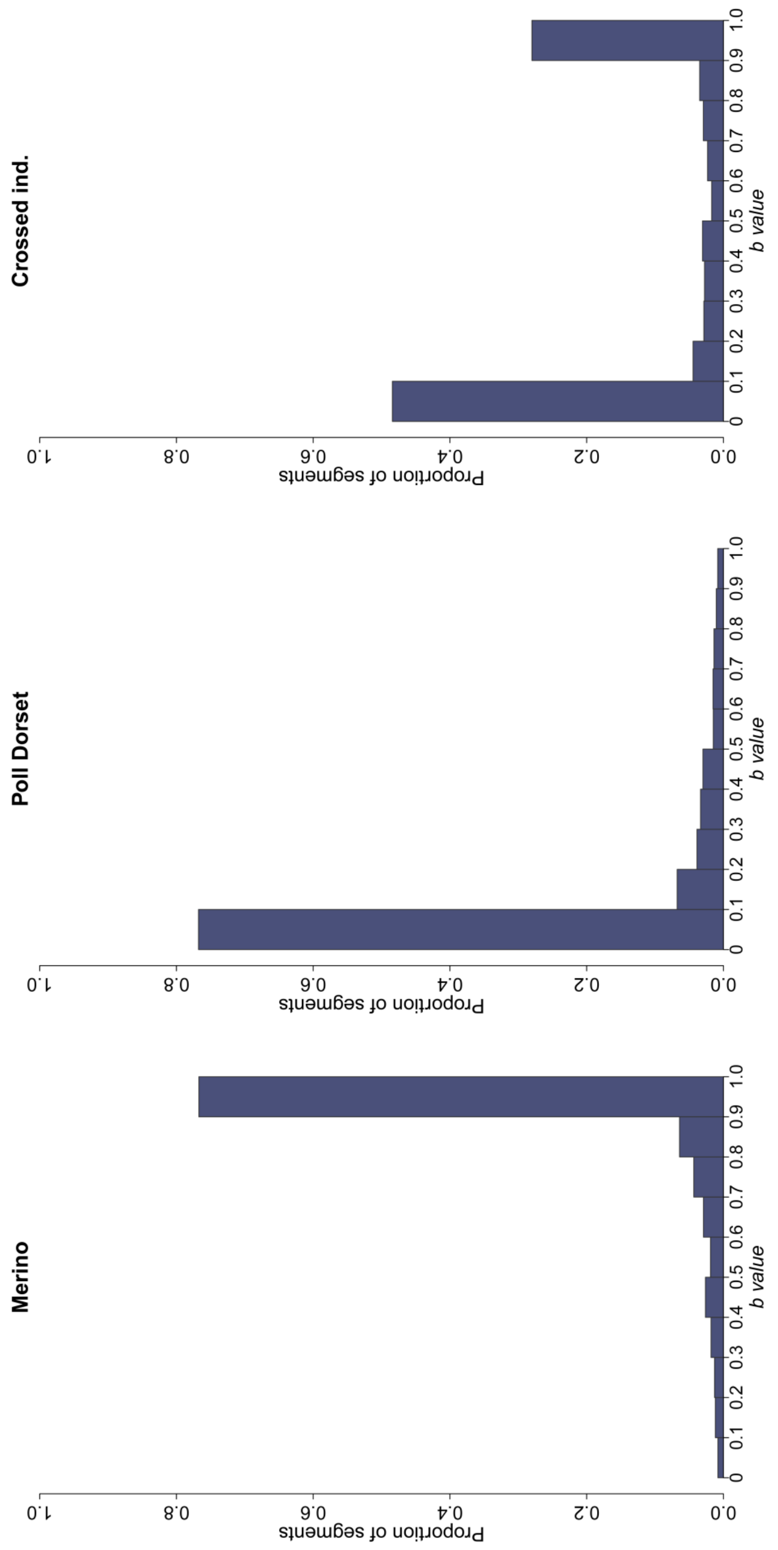

Fig. S3. Proportion of *Merino recovery* (upper panels) after one or five generations of management and *ΔF* (lower panels) after one or five (cumulative *ΔF)* generations of management using the GBLUP approach, for 1, 3 or 5 generations of admixture (20 individuals contributing).

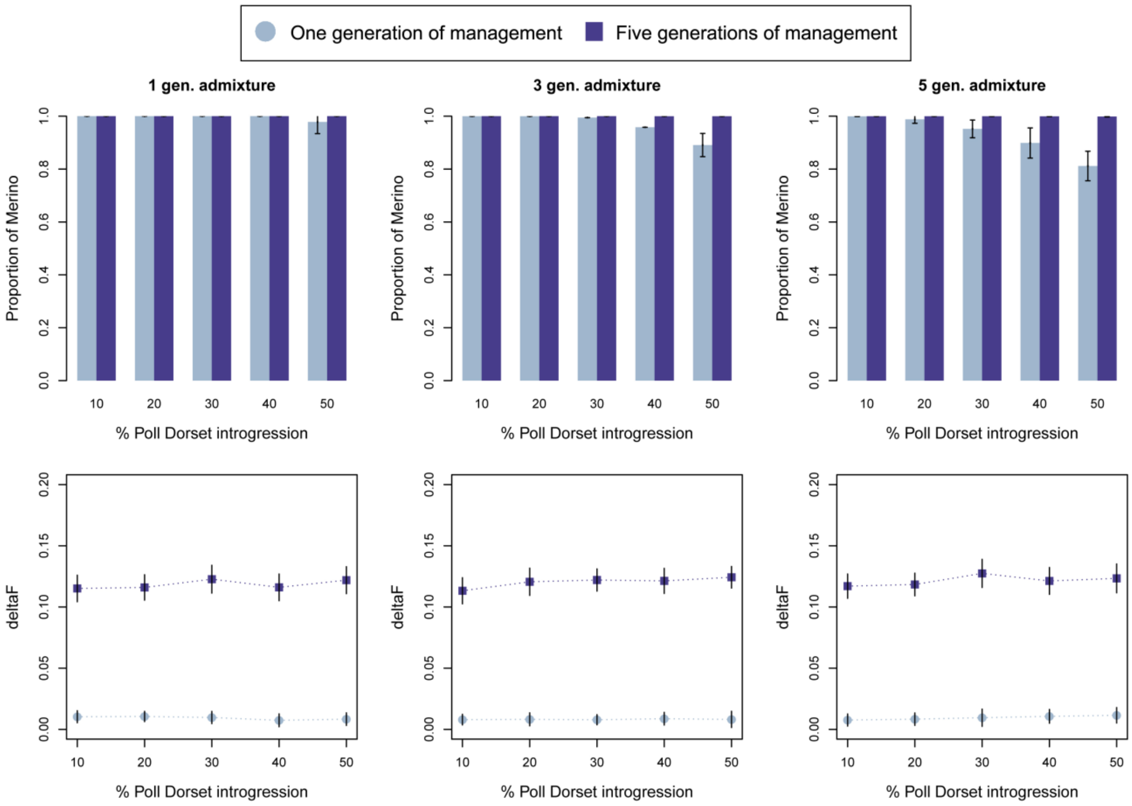

Fig. S4. Comparison of the GBLUP solutions and the haplotypes results in the crossed individuals. The GBLUP solutions are represented in the vertical axis and the mean *b* values per individual obtained in the haplotype approach in the horizontal axis.

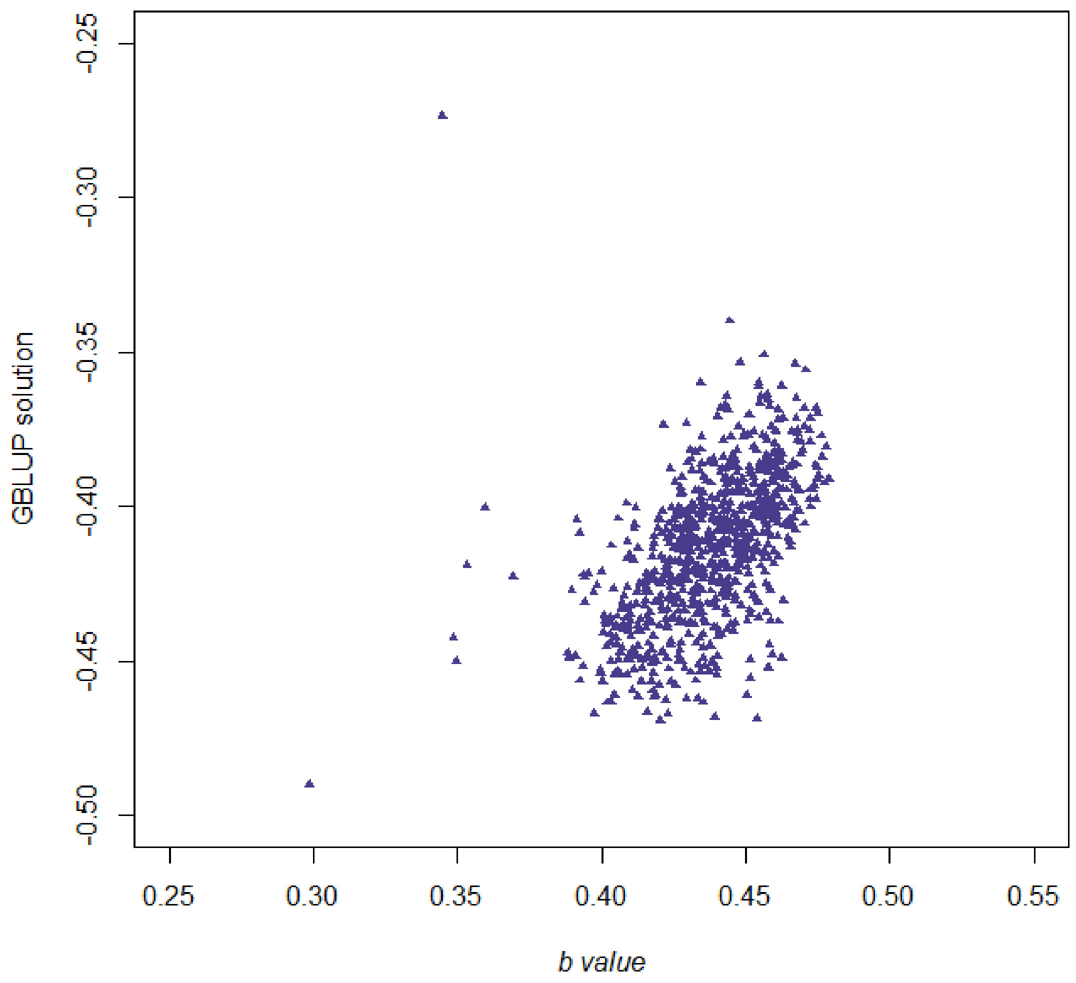

Table S1.

Proportion of *Merino recovery* and *ΔF* obtained after one generation of de-introgression in scenarios with 20 generations of admixture (40 individuals contributing) using the GBLUP approach with a reference population of 188 Poll Dorset and 20 F1 crossed individuals from the original population.

	**% Poll Dorset introgression**				
	**10**	**20**	**30**	**40**	**50**
Merino Recovery	0.950 ± 0.031	0.864 ± 0.059	0.793 ± 0.074	0.715 ± 0.104	0.607 ± 0.096
ΔF	0.007 ± 0.004	0.006 ± 0.005	0.007 ± 0.004	0.008 ± 0.005	0.006 ± 0.006

Table S2.

Proportion of *Merino recovery* and *ΔF* obtained after one generation of de-introgression in scenarios with 20 generations of admixture (40 individuals contributing) using Structure (linkage model for K=2 and default options).

	**STRUCTURE**				
**% Poll Dorset**	**10**	**20**	**30**	**40**	**50**
Merino Recovery	0.918 ± 0.034	0.834 ± 0.053	0.771 ± 0.071	0.698 ± 0.103	0.588 ± 0.095
ΔF	0.006 ± 0.006	0.006 ± 0.005	0.006 ± 0.004	0.006 ± 0.004	0.007 ± 0.004
